# Supplementary material for: Heat adaptation of phage T7 under an extended genetic code
Source: Virus Evol. 2021 Dec 1;7(2):veab100. doi: 10.1093/ve/veab100 (PMC8923235; doi:10.1093/ve/veab100)
Supplement: veab100_Supp [file veab100_supp.zip › Sequencing_Analysis.zip!/Sequencing_Analysis/A1/evidence/JC_SIDE_2_59.html]

BRESEQ :: Evidence


| New junction evidence | | | | | | | | | | | |
| --- | --- | --- | --- | --- | --- | --- | --- | --- | --- | --- | --- |
|  | | seq id | position | reads (cov) | reads (cov) | score | skew | freq | annotation | gene | product |
| \* | ? | NC\_001604.1 | = 13455 | 684 (0.220) | 103 (0.030) | 80/586 | 17.8 | 20.7% | intergenic (–/–) | *–/–* | –/– |
|  | ? | NC\_001604.1 | 14331 = | 121 (0.040) |  |  |  |  | intergenic (–/–) | *–/–* | –/– |
| Rejected: Coverage evenness skew score above cutoff. | | | | | | | | | | | |

Only 100 of 774 total aligned reads displayed.  

`AAGAACTTAGGCTGGACTTGGACAAGACAGACTGGCTGGAAGAAACCAGAAGTTACCAAGCTGAGTCATAAGGTGTTCGATAAGGACACTATGACCCACATCAAGGCTGGTGATTGGGTTAAGGTTGACATGGGAGTTGTTGGTGGATACGGCTACGTCCGCTCAGTTAGTGGCAAATATGCACAAGTGTCATACATCACAGGTGTTACTCCACGCGGTGCAATCGTTGCCGATAAGACCAACATGATTCACACAGGTTTCTTGACAGTTGTTTCATATGAAGAGATTGTTAAGTCACGATAATC‑AATAGGAGAAATCAATATGATCGTTTCTGACATCGAAGCTAACGCCCTCTTAGAGAGCGTCACTAAGTTCCACTGCGGGGTTATCTACGACTACTCCACCGCTGAGTACGTAAGCTACCGTCCGAGTGACTTCGGTGCGTATCTGGATGCGCTGGAAGCCGAGGTTGCACGAGGCGGTCTTATTGTGTTCCACAACGGTCACAAGTATGACGTTCCTGCATTGACCAAACTGGCAAAGTTGCAATTGAACCGAGAGTTCCACCTTCCTCGTGAGAACTGTATTGACACCCTTGTG  >  NC_001604.1/14032‑14631`  

                                                                                                                                                                                                                                                                                                           `|`                                                                                                                                                                                                                                                                                                               
`tactgacctacgtggttctggcgcactacgccaactatctgatactattattgcccttgagcgtaatcagcaaggcgatatgcctaaccttgtcctcgttcgtattctcaagtgccgctttactggtgatactggtatcgctggctacatggaatacaacaaggaaaccggatggcttgaaccatcaagttactcaggggaagaagagtcacactcagagtcaacagactggtccaacgacactgacttctgacaggattcttgaCAGTTGTTTCATATGAAGAGATTGTTAAGTCACGAt                                                                                                                                                                                                                                                                                                              <  1:19197‑M2/36‑1 (MQ=255)`  
 `ggaagaagagtcacactcagagtcaacagacTGGCTGGAAGAAACCAGAAGTTACCAAGCTGAGTCATAAGGTGTTCGATAAGGACACTATGACCCACATCAAGGCTGGTGATTGGGTTAAGGTTGACATGGGAGTTGTTGGTGGATACGGCTACGTCCGCTCAGTTAGTGGCAAATATGCACAAGTGTCATACATCACAGGTGTTACTCCACGCGGTGCAATCGTTGCCGATAAGACCAACATGATTCACACAGGTTTCTTGACAGTTGTTTCATATGAAGAGATTGTTAAGTAACGATa                                                                                                                                                                                                                                                                                                             <  1:447558/276‑1 (MQ=255)`  
    `gacctacgtggttctggcgcactacgccaactacctgatactattattgcccttgagcgtaatcagcaaggctatatgcctaaccttgtcctcgttcgtattctcaagtgccgctttactggtgatactgttatcgctggctacatgtactacaacaaggaaaccggatggcttgaaccatcaagttactcaggggaagaagagtcacactcagagtcaacagactggtccaacgacactgacttctgataggatccttgaCAGTTGTTTCATATGAAGAGATTGTTAAGTCACGATAATc                                                                                                                                                                                                                                                                                                          >  1:40500‑M2/262‑301 (MQ=255)`  
        `tacgtggttctggcgcactacgccaactatctgatactattattgcccttgagcgtaatcagcaaggcgatatgcctaaccttgtcctcgttcgtattctcaagtgccgctttactggtgatactggtatcgatggctacatggaatacaacaaggaaaccggatggcttgaaccatcaagttactcaggggaagaagagtcacactcagagtcaacagactggtccaacgacactgacttctgacaggattcttgaCAGTTGTTTCATATGAAGAGATTGTTAAGTCACGATAATC‑AATa                                                                                                                                                                                                                                                                                                     >  1:214248‑M2/258‑301 (MQ=255)`  
              `gttctggcgcactacgccaactatctgatactattattgcccttgagcgtaatcagcaaggcgatatgcctaaccttgtcctcgttcgtattctcaagtgccgctttactgttgatactggtatcgcaggctgcatggaatacaacaaggaaaccggatggcttgaaccatcaagttactcaggggaagaagagtcacactcagagtcaacagactggtccaacgacactgacttctgacaggattcttgaCAGTTGTTTCATATGAAGAGATTGTTACGTCACGATAATC‑AATAGGAGaa                                                                                                                                                                                                                                                                                               >  1:61366‑M2/252‑301 (MQ=255)`  
              `gttctggcgcactacgccaactatctgatactattattgcccttgagcgtaatcagcaaggcgatatgcctaaccttgtcctcgttcgtattctcaagtgccgctttactggtgatactggtatcgctggctacatggaatacaacaaggaaaccggatggcttgaaccatcaagttactcaggggaagaagagtcacactcagagtcaacagactggtccaacgacactgacttctgacaggattcttgaCAGTTGTTTCATATGAAGAGATTGTTAAGTCACGATAATC‑AATAGGAGaa                                                                                                                                                                                                                                                                                               >  1:251744‑M2/252‑301 (MQ=255)`  
                   `ggcgcactacgccaactatctgatactattattgcccttgagcgtaatcagcaaggcgatatgcctaaccttgtcctcgttcgtattctcaagtgccgctttactggtgatactggtatcgctggctacatggaatacaacaaggaaactggatggcttgaaccatcaagttactcaggggaagaagagtcacactcagagtcaacagactggtccaacgacactgacttctgacaggattcttgaCAGTTGTTTCATATGAAGAGATTGTTAAGTCACGATAATC‑AATAGGAGAAATCaa                                                                                                                                                                                                                                                                                          >  1:388930‑M2/247‑301 (MQ=255)`  
                       `cactacgccaactatctgatactattattgcccttgagcgtaatcagcaaggcgatatgcctaaccttgtcctcgttcgtattctcaagtgccgctttactggtgatactggtatcgctggctacatggaatacaacaaggaaaccggatggcttgaacaatcaagttactcaggggaagaagagtcacactcagagtcaacagactggtccaacgacactgacttctgacaggatacttgaCAGTTGTTTCATATGAAGAGATTGTTAAGTCACGATAATC‑AATAGGAGAAATCaatata                                                                                                                                                                                                                                                                                      >  1:84834‑M2/243‑300 (MQ=255)`  
                           `acgccaactatctgatactatgatggcgtttgagcgtacgcagcagggctatatgactaactttgtcctcgttcggcttatcaagtgcagctttactgatgatactggtatctctggctacatgaaatacaacaaggaaccctgatggatggaaccataaagttactcaggggaagaagtgacacactcagagtcaacagactggtccaacgacactgacttctgacaggattcttgaCAGTTGTTTCATATGAAGAGATTGTTAAGTCACGATAATC‑AATAGGAGAAATCAATATGATCg                                                                                                                                                                                                                                                                                  <  1:420392‑M2/63‑1 (MQ=255)`  
                                  `ctatctgatactattattgcccttgagcgtaatcagcaaggcgatatgcctaaccttgtcctcgttcgtattctcaagtgccgctttactggtgatactggtatcgctggctacatggaatacaacaaggaaaccggatggcttgaaccatcaagttactcaggggaagaagagtcacactcagagtcaacagactggtccaacgacactgacttctgacaggattcttgaCAGTTGTTTCATATGAAGAGATTGTTAAGTCACGATAATC‑AATAGGAGAAAACATTATGATCGTTTCTGa                                                                                                                                                                                                                                                                           >  1:226966‑M2/232‑301 (MQ=255)`  
                                   `aagagacagctattattgccctttatcgtaatcagcaaggcgatattcctaaccttgtcctcgttcgtattctcaagtgccgctttactggtgatactggtatcgctggctacatggaatacaacaaggaaaccggatggcttgaaccatcaagttactcaggggaagaagagtcacactcagagtcaacagactggtccaacgacactgacttctgacaggattcttgaCAGTTGTTTCATATGAAGAGATTGTTAAGTCACGATAATC‑AATAGGAGAAATCAATATGATCGTTTCTGAc                                                                                                                                                                                                                                                                          <  1:247878‑M2/71‑1 (MQ=255)`  
                                    `taagagacagattattgcccttgagcgtaatcagcaaggcgatatgcctaaccttgtcctcgttcgtattctcaagtgccgctttactggtgatactggtatcgctggctacatggaatacaacaaggaaaccggatggcttgaaccatcaagttactcaggggaagaagagtcacactcagagtcaacagactggtccaacgacactgacttctgacaggattcttgaCAGTTGTTTCATATGAAGAGATTGTTAAGTCACGATAATCAAACAGGAGAAATCAATATGATCGTTTCTGAc                                                                                                                                                                                                                                                                          <  1:477915‑M2/72‑1 (MQ=255)`  
                                              `attattgcccttgagcgtaatcagcaaggcgatatgcctaaccttgtcctcgttcgtattctcaagtgccgctttactggtgatactggtatcgctggctacatggaatacaacaaggaaaccggatggcttgaaccatcaagttactcaggggaagaagagtcacactcagagtcaacagactggtccaacgacactgacttctgacaggattcttgaCAGTTGTTTCATATGAAGAGATTGTTAAGTCACGATAATC‑AATAGGAGAAATCAATATGATCGTTTCTGACATCGAAGCTaa                                                                                                                                                                                                                                                               >  1:136830‑M2/220‑301 (MQ=255)`  
                                              `attattgcccttgagcgtaatcagcaaggcgatatgcctaaccttgtcctcgttcgtattctcaagtgccgctttactggtgatactggtatcgctggcgacatggaatacaacaaggaaaccggatggcttgaaccatcaagttactcaggggaagaagagtcacactcagagtcaacagactggtccaacgacactgacttctgacaggattcttgaCAGTTGTTTCATATGACGAGATTGTTAAGTCACGATAATC‑AATAGGAGAAATCAATATGATCGTTTCTGACATCGAAGCTaa                                                                                                                                                                                                                                                               >  1:260432‑M2/220‑301 (MQ=255)`  
                                              `attattgcccttgagcgtaatcagcaaggcgatatgcctaaccttgtcctcgttagtattctcaagtaccgctttactggtgatactggtatcgctggctacatggaatacaacaaggaaaccggatggcttgaaccatcaagttactcaggggaagaagagtcacactcagagtcaacagactggtccaacgacactgacttctgacaggattcttgaCAGTTGTTTCATATGAAGAGATTGTTAAGtcacgataatc‑aataggagaaatcaatactgtctcttatacacatctccgagc                                                                                                                                                                                                                                                               >  1:382150‑M2/220‑276 (MQ=255)`  
                                                `tatgtcccttgagcgtaatcagcaaggcgatatgcctaaccttggcctcgttcgtattctcaagtgccgctttactggggacactggtatcgctggctacatggaatacaacaaggaaaccggatggcttgaaccatcaatttactcaggggaagaagagtcacactcagagtcaacagactggtccaacgacactgacttctgacaggattcttgaCAGTTGTTTCATATGAAGAGATTGTTAAGTCACGATAATC‑AATAGGAGAAAGCAATATGATCGTTTCTGACATCGAAGCTAACg                                                                                                                                                                                                                                                             <  1:399324‑M2/84‑1 (MQ=255)`  
                                                  `ttgcccttgagcgtaatcagcaaggcgatatgcctaaccttgtcctcgttcgtattctcaagtgccgctttactggtgatactggtatcgctggctacatggaatacaacaaggaaaccggatggcttgaaccatcaagttactcaggggaagaagagtaacactcagagtcaacagactggtccaacgacactgacttctgacaggattcttgaCAGTTGTTTCATATGAAGAGATTGTTAAGTCACGATAATC‑AATAGGAGAAATCAATATGATCGTTTCTGACATCGAAGCTAACGcc                                                                                                                                                                                                                                                           >  1:8194‑M2/216‑301 (MQ=255)`  
                                                      `ccttgagcgtaatcagcaaggcgatatgcctaaccttgtcctcgttcgtattctcaagtgccgctttactggtgatactggtatcgctggctacatggaatacaacaaggaaaccggatggcttgaaccatctagttactcaggggaagaaaagtcacactcagagtcaacagactggtccaacgacactgacttctgacaggattcttgaCAGTTGTTTCATATGAAGAGCTTGTTAAGTCACGATTATC‑AATAGGCAAAATCAATATGATCGTTTCTGACATCGAAGCTAACGCCctct                                                                                                                                                                                                                                                       >  1:188045‑M2/212‑301 (MQ=255)`  
                                                         `taagcataatcagcaaggcgagatgcctaaccttgtcctcgttcgtagtaccaagtgccactttactggtgagactggtatcgctggctacatggaatacaacaaggaaaccggatggcttgaaccatcaagttactcaggggaagaagagtcacactcagagtcaacagactggtccaacgacactgacttctgacaggattcttgaCAGTTGTTTCATATGAAGAGATTGTTAAGTCACGATAATC‑AATAGGAGAAATCAATATGATCGTTTCTGACATCGAAGCTAACGCCCTCTTag                                                                                                                                                                                                                                                    <  1:32577‑M2/93‑1 (MQ=255)`  
                                                           `gggctcggagatgtgtataagagacagaccttgtcctcgttcgtattctcaagtgccgctttactggtgatactggtatcgctggctacatggaatacaacaaggaaaccggatggcttgaaccatcaagttactcaggggaagaagagtcacactcatagtcaacagactggtccaacgacactgacttctgacaggattcttgaCAGTTGTTTCATATGAAGAGATTGTTAAGTCACGATAATC‑AATAGGAGAAATCAATATGATCGTTTCTGACATCGAAGCTAACGCCCTCTTagag                                                                                                                                                                                                                                                  <  1:124041‑M2/95‑1 (MQ=255)`  
                                                           `gagacagtcagcaaggcgatatgcctaaccttgtcctcgttcgtattctcaagtgccgctttactggtgatactggtatcgctggctacatggaatacaacaaggaaaccggatggcttgaaccatcaagttactcaggggaagaagagtcacattcagagtcaacagactggtccaacgacactgacttctgacaggattcttgaCAGTTGTTTCATATGAAGAGATTGTTAAGTCACGATAATC‑AATAGGAGAAATCAATATGATCGTTTCTGACATCGAAGCTAACGCCCTCTTagag                                                                                                                                                                                                                                                  <  1:30543‑M2/95‑1 (MQ=255)`  
                                                             `agatgtgtataagagacagggcctaaccttgtcctcgttcgtattctcaagtgccgctttactggtgatactggtatcgctggctacatggaataaaaaaaggaaaccggatggcttgaaccatcaagttactcaggggaagaagagtcacactcagagtcaacagactggtccaacgacactgaattctgacaggagtcttgaCAGTTGTTTCATATGAAGAGATTGTTAAGTCACGATAATC‑AATAGGAGAAATCAATATGATCGTTTCTGACATCGAAGCTAACGCCCTCTTagagag                                                                                                                                                                                                                                                <  1:113334‑M2/97‑1 (MQ=255)`  
                                                              `gtaatcagcaaggcgatatgcctaaccttgtcctcgttcgtattctcaagtgccgctttactggtgatactggtatcgctggctacatggaatacaacaaggaaaccggatggcttgaaccatcaagttactcaggggaagaagagtcacactcagagtcaacagactggtccaacgacactgacttctgacagaattcttgaCAGTCGTTTCATATGAAGAGATTGTTAAGTAAAGATAATC‑AATAGGAGCAATCAATCTGATCGTTTCTGACATCGAAGCTAAcgccctcttcgctgtc                                                                                                                                                                                                                                               >  1:465740‑M2/204‑294 (MQ=255)`  
                                                                   `cagcaaggcgatatgcctaaccttgtcctcgttcgtattctcaagtgccgctttactggtgatactggtctcgctggctacatggaatacaacaaggaaaccggatggcttgaaccatcaagttactcaggggaagaagagtcacactcagagtcaacagactggtccaacgacactgacttctgacaggattcttgaCAGTTGTTTCATATGAAGAGATTGTTAAGTCACGATAATC‑ATTAGGAGAAATCAATATGATCGTTTCTGACATCGAAGCTAACGCCCTCTTAGAGAGCGTCAc                                                                                                                                                                                                                                          <  1:1641‑M2/103‑1 (MQ=255)`  
                                                                          `gtgacaggcctaaccttgtcctcgttcggattctcaagtgccgctttacttgtgatacttgtatcgctggctacatggaatacaacaatgaaaccggatggcttcaaccatcaagttactcaggggaagaagagtcaccctcagagtcaacagactggtccaacgacactgacttctgacaggattcttgaCAGTTGTTTCATATGAAGAGATTGTTTAGTCACGATAATC‑AATAGGAGAAATCAATATGATCGTTTCTGACATCGAAGCTAACGCCCTCTTAGAGAGCGTCACTAAGTTc                                                                                                                                                                                                                                   <  1:354349‑M2/110‑1 (MQ=255)`  
                                                                           `ttCGATAAGGACACTATGACCCACATCAAGGCTGGTGATTGGGTTAAGGTTGACATGGGAGTTGTTGGTGGATACGGCTACGTCCGCTCAGTTAGTGGCAAATATGCACAAGTGTCATACATCACAGGTGTTACTCCACGCGGTGCAATCGTTGCCGATAAGACCAACATGATTCACACAGGTTTCTTGACAGTTGTTTCATATGAAGAGATTGTTAAGTCACGATAATC‑AATAGGAGAAATCAATATGATCGTTTCTGACATCGAAGCTAACGCCCTCTTAGAGAGCGTCACTAAGTTcc                                                                                                                                                                                                                                  <  1:4919/301‑1 (MQ=255)`  
                                                                           `cgatatgcctaaccttgtcctcgttcgtattctcaagtgcctctttactggtgatactggtatcgctggctacatggaatacaacaaggaaaccggatggctttaaccatcaagttactcaggggaagaagagtcaccctcagagtcaacagactggtccaacgacacttacttctgacaggattcttgaCAGTTGTTTCATATGAAGAGATTGTTAAGTCACGATAATC‑AATAGGAGAAATCAATATGATCGTTTCTGACATCGAAGCTAACGCCCTCTTAGAGAGCGTCACTAAGTTcc                                                                                                                                                                                                                                  >  1:243491‑M2/191‑301 (MQ=255)`  
                                                                                `tgcctaaccttgtcctcgttcgtattctcaagtgccgctttactggtgatactggtatcgctggctacatggaatacaacaaggaaaccggatggcttgaaccatcaagttactcaggggaagaagagtcacactcagagtcaacagactggtccaacgacactgacttctgacaggattcttgaCAGTTGTTTCATATGAAGAGATTGTTAAGTCACGATAATC‑AATAGGAGAAATCAATATGATCGTTTCTGACATCGAAGCtaacgccctcttagagagctgtctcttatacacatct                                                                                                                                                                                                                             >  1:423459‑M2/186‑283 (MQ=255)`  
                                                                                 `gcctaaccttgtcctcgttcgtattctcaagtgccgctttactggtgatactggtatcgctggctacatggaatacaacaaggaaaccggatggcttgaaccatcaagttactcaggggaagaagagtcacactcagagtcaacagactggtccaacgacactgacttctgacaggattcttgaCAGTTGTTTCATATGAAGAGATTGTTAAGTAACGATAATCAAACAGGAGAAATAAATATGATCGTTTCTTACATCGAAGCTAACGCACTCTTAGCGATCCTCAATAAGTTCAACTGc                                                                                                                                                                                                                             >  1:174838‑M2/185‑301 (MQ=255)`  
                                                                                               `tcgttcgtattctcaagttccgctttactggtgatactggtatcgctggctacatggaatacaacaaggaaaccggatggcttgatccatcaagttactcaggggaagaagagtcacactcagagtcaacagactggtccaacgacactgacttctgacagtattcttgaCAGTTGTTTCATATGAAGAGATTGTTAAGTCACGATAATC‑AATAGGAGAAATCAATATGATCGTTTCTGACATCGAAGCTAACGCCCTCTTAGAGAGCGTCACTAAGTTCCACTGCGGGGTTATCTACGac                                                                                                                                                                                                              <  1:159383‑M2/131‑1 (MQ=255)`  
                                                                                                `cgttcgtattctcaagtgccgctttactggtgatactggtatcgctggctacatggaatacaacaaggaaaccggatggcttgaaccatcaagttactcaggggaagaagagtcacactcagagtcaacagactggtccaacgacactgacttctgacaggattcttgaCAGTTGTTTCATATGAAGAGATTGTTAAGTCACGATAATC‑AATAGGAGAAATCAATATGATCGTTTCTGACATCGAAGCTAACGCCCTCTTAGAGAGCGTCACTAAGTTCCACTGCGGGGTTATCTACGact                                                                                                                                                                                                             >  1:443879‑M2/170‑301 (MQ=255)`  
                                                                                                  `tttgtattcgcaagtgccgcattaatgggtataatggtatcggtggctacatggaatacaacaaggaaaccggagggcttgaaccatcaatttactcaggggaagaagagtcacactcagagtcaacagactggtccaacgacactgacttctgacaggattcttgaCAGTTGTTTCATATGAAGAGATTGTTAAGTCACGATAATC‑AATAGGAGAAATCAATATGATCGTTTCTGACATCGAAGCTAACGCCCTCTTAGAGAGCGTCACTAAGTTCCACTGCGGGGTTATCTACGactac                                                                                                                                                                                                           <  1:449319‑M2/134‑1 (MQ=255)`  
                                                                                                     `ttgtataagtgccaggctttactggtgatactggtatcgcgggctacatggaatacaacaaggaaaccggatggcgtgaaccatcaagttactcaggggaagaagagtcacactcagagtaaacagactggtccaacgacactgacatctgacaggattcttgaCAGTTGTTTCATATGAAGAGATTGTTAAGTCACGATAATC‑AATAGGAGAAATCAATATGATAGTTTCTGACATCGAAGCTAACGCCCTCTTAGAGAGCGTCACTAAGTTCCACTGCGGGGTTATCTACGACTACTcc                                                                                                                                                                                                        <  1:16756‑M2/137‑1 (MQ=255)`  
                                                                                                       `attctcaagtgccgatttactggtgatactggtatcgctggctacatggaatacaacaaggaaaccggatggcttgaaccatcaagttactcaggggaagaagagtcacactcagagtcaacagactggtccaacgacactgacttctgacaggattcttgaCAGTTGTTTCATATGAAGAGATTGTTAAGTCACGATAATC‑AATAGGAGAAATAAATATGATCGTTTCTGACATCGAAGCTAACGCCCTCTTAGAGAGCGTCACTAAGTTCCACTGCGGGGTtatctacgacctgtctct                                                                                                                                                                                                      >  1:95196‑M2/163‑293 (MQ=255)`  
                                                                                                       `attatcaagtgccgctttactggtgatactggtatcgatggctacatggaagacaacaaggaaaccggatggcttgaaccatcaagttactcaggggaagaagagtcacactcagagtcaacagactggtccaacgacacggacttctgacaggattcttgaCAGTTGTTTCATATGAAGAGATTGTTAAGTCACGATAATC‑AATAGGAGAAATCAATATGATCGTTTCTGACATCGAAGCTAACGCCCTCTTAGAGAGCGTCACTAAGTTCCACTGCGGGGTTATCTACGACTACTCCAc                                                                                                                                                                                                      <  1:378468‑M2/139‑1 (MQ=255)`  
                                                                                                          `ctcaagtgccgctttactggtgatactggtatcgctggctacatggaatacaacaaggaaaccggatggcttgaaccatcaagttactcaggggaagaagagtcacactcagagtcaacagactggtccaacgacactgacttctgacaggattcttgaCAGTTGTTTCATATGAAGAGAGTGTTAAGTCACGATAATC‑AATAGGAGAAATCAATATGATCGTTTCTGACATCGAAGCTAACGCCCTCTTAGAGAGCGTCACTAAGTTCCACTGCGGGGTTATCTACGACTACTCCACCGc                                                                                                                                                                                                   <  1:463000‑M2/142‑1 (MQ=255)`  
                                                                                                          `ctcaagtgccgctttactggtgatactggtatcgctggctacatggaatacaacaaggaaaccggatggcttgaaccatcaagttactaaggggaagaagagtcacactcagagtcaacagactggtccaacgacactgacatctgacaggattcttgaCAGTTGTTTCATATGAAGAGATTGTTAAGTCACGATAATC‑AATAGGAGAAATCAATATGATCGTTTCTGACATCGAAGCTAACGCCCTCTTAGAGAGCGTCACTCAGTTCCACTGCGGGGTTATCTACGACTACTACACcga                                                                                                                                                                                                   >  1:53843‑M2/160‑300 (MQ=255)`  
                                                                                                               `gtgccgctttactggtgatactggtatcgctggctacatggaatacaacaaggaaaccggatggcttgaaccatcaagttactcaggggaagaagagtcacactcagagtcaacagactggtccaacgacactgacttctgacaggattcttgaCAGTTGTTTCATATGAAGAGATTGTTAAGTCACGATAATC‑AATAGGAGAAATCAATATGATCGTTTCTGACATCGAAGCTAACGCCCTCTTAGAGAGCGTCACTAAGTtccactgcggggttatctactgtctcttataccaatctc                                                                                                                                                                                              >  1:127260‑M2/155‑282 (MQ=255)`  
                                                                                                                   `gggTTAAGGTTGACATGGGAGTTGTTGGTGGATACGGCTACGTCCGCTCAGTTAGTGGCAAATATGCACAAGTGTCATACATCACAGGTGTTACTCCACGCGGTGCAATCGTTGCCGATAAGACCAACATGATTCACACAGGTTTCTTGACAGTTGTTTCATATGAAGAGATTGTTAAGTCACGATAATC‑AATAGGATAAATCAATATGATCGTTTCTGACATCGAAGCTACCGCCCTCTTAGAGAGCGTCACTAAGTTCCACTGCGGGGTTATCTACGACTACTCCACCGCTGAGTACGt                                                                                                                                                                                          >  1:272662/1‑301 (MQ=255)`  
                                                                                                                    `gctttactggtgatactggtatcgctggctacatggaatacaacaaggaaaccggaaggcttgaaccatcaagttactcaggggaagaagagtcacactcagagtcaacagactggtccaacgacactgacttctgacaggattcttgaCAGTTGTTTCATATGAAGAGATTGTTAAGTCACGATAATC‑AATAGGAGAAATCAATATGATCGTTTCTGACATCGAAGCTAACGCCCTCTTAGAGAGCGTCACTAAGTTCCACTGCGGGGTTATCTACGACTACTCCACCGCTGAGTACGTa                                                                                                                                                                                         <  1:58541‑M2/152‑1 (MQ=255)`  
                                                                                                                          `tcggagatgtgtataagagacagtacatggaatacaacaaggaaaccggatggcttgaaccatcaagttactcaggggaagaagagtcacactcagagtcaacagactggtccaacgacactgacttctgacaggattcttgaCAGTTGTTTCATATGAAGAGATTGTTAAGTCACGATAATC‑AATAGGAGAAATCAATATGATCGTTTCTGACATCGAAGCTAACGCCCTCTTAGAGAGCGTCACTAAGTTCCACTGCGGGGTTATCTACGACTACTCCACCGCTGAGTACGTAAGCTAc                                                                                                                                                                                   <  1:377043‑M2/158‑1 (MQ=255)`  
                                                                                                                          `ggggtatactggtatcgctggctacatggaatacaacaaggaaaccggatggcttgaaccatcaagttactcagggggaagaagagtcacactcatagtcaacagactggtccaatgacactgacttctgacaggattcttgaCAGTTGTTTCATATGAAGAGATTGTTAAGTCACGATAATC‑AATAGGAGAAATCAATATGATCGTTTCTGACATCGAAGCTAACGCCCTCTTAGAGAGCGTCACTAAGTTCCACTGCGGGGTTATCTACGACTACTCCACCGCTGAGTACGTAAGCTAc                                                                                                                                                                                   <  1:209200‑M2/158‑1 (MQ=255)`  
                                                                                                                          `aaggtgatactggtatcgctggctacatggaatacaacaaggaaaccggatggcttgaaccatcaagttactcaggggaagaagagtcacactcagagtcaacagactggtccaacgacactgccttctgacaggattcttgaCAGTTGTTTCATATGAAGAGATTGTTAAGTCACGATAATC‑AATAGGAGAAATCAATATGATCGTTTCTGACATCGAAGCTAACGCCCTCTTAGAGATCGTCACTAAGTTCCATTGCGGGGTTATCACAGACTACTCCACCCCTGAgtacgtactgttt                                                                                                                                                                                   >  1:337825‑M2/144‑295 (MQ=255)`  
                                                                                                                                         `ggctcggagatgtgtataagagacaggaatacaacaaggaaaccggatggcttgaaccatcaagttactcaggggaagaagagtcacactcagagtcaacagactggtccaacgacactgacttctgaCAGTTGTTTCATATGAAGAGATTGTTAAGTCACGATAATC‑AATAGGAGAAATCAATATGATCGTTTCTGACATCGAAGCTAACGCCCTCTTAGAGAGCGTCACTAAGTTCCACTGCGGGGTTATCTACGACTACTCCACCGCTGAGTACGTAAGCTACCGTCCGAGTGACTTc                                                                                                                                                                    <  1:475036‑M2/173‑1 (MQ=255)`  
                                                                                                                                          `cgctggctacatggaatacaacaaggaaaccggatggcttgaaccatcaagttactcaggggaagaagagtcacactcagagtcaacagactggtccaacgacactgacttctgacaggattcttgaCAGTTGTTTCATATGAAGAGATTGTTAAGTCACGATAATC‑AATAGGAGAAATCAATATGATCGTTTCTGACATCGAAGCTAACGCCCTCTTAGAGAGCGTCACTAAGTTCCACTGCGGGgttatctacgactactccaccgctgagtccctgtctcttatccacatctccgagc                                                                                                                                                                   >  1:319562‑M2/128‑274 (MQ=255)`  
                                                                                                                                                   `catggaatacaacaaggaaaccggatggcttgaaccatcaagttactcaggggaagaagagtcacactcagagtaaacagactggtccaacgacactgacttctgacaggattcttgaCAGTTGTTTCATATGAAGAGATTGTTAAGTCACGATAATC‑AATAGGAGAAATCAATATGATCGTTTCTGACATCGAAGCTAACGCCCTCTTAGAGAGCGTCACTAAGTTCCACTGCGGGGTTATCTACGACTACTCCACCGCTGAGTACGTAAGCTACCGTCCGAGTAACTTcggtgctgtct                                                                                                                                                          >  1:203886‑M2/119‑296 (MQ=255)`  
                                                                                                                                                   `aatggaatacaacaaggaaaacggatgggttgaaccatcaagtaactcagtagaagaagagtcacactcagagaaaacagacagatccaaagaaactgacttctgacaggattcttgaCAGTTGTTGCATATGAAGAGATGGTGAAGTAACGATAATC‑AATAGTAGAAATCAATATAATCGTTTCTGAAATCGAAGATAACGCCATCTTAGAGAGCGTCACTAAGTTCCACTGCGGGGTTATCTACGACTACTCCACCGCTGAGTATGTAAGCTACCGTCCGAGTGACTTCGGTGCGTATc                                                                                                                                                          <  1:88311‑M2/183‑1 (MQ=255)`  
                                                                                                                                                       `gaatacaacaaggaaaccggatggcttgaaccatcaagttactcaggggaagaagagtcacactcagagtcaacagactggtccaacgacactgacttctgacaggattcttgaCAGTTGTTTCATATGAAGAGATTGTTAAGTCACGATAATC‑AATAGGAGAAATCAATATGATCGTTTCTGACATCGAAGCTAACGCCCTCTTAGAGAGCGTCACTAAGTTCCACTGCGGGGTTATCTACGACTACTCCACCGCTGAGTACGTAAGCTACCGTCCGAGTGACTTCGGTGCGTATCTGGa                                                                                                                                                      >  1:26456‑M2/115‑301 (MQ=255)`  
                                                                                                                                                        `gtgtataagagacagccggatgtcttgaaccatcaagttactcaggggaagaagagtcacactcagagtcaacagactggtccaacgacactgacttctgacaggattctttaCAGTTGTTTCATATGAAGAGATTGTTAAGTCACGATAATC‑AATAGGAGAAATCAATATGATCGTTTCTGACATCGAAGCTAACGCCCTCTTAGAGAGCGTCACTAAGTTCCACTGCGGGGTTATCTACGACTACTCCACCGCTGAGTACGTAAGCTATCGTCCGAGTGACTTCGGTGCGTATCTGGAt                                                                                                                                                     <  1:32848‑M2/188‑1 (MQ=255)`  
                                                                                                                                                         `aagagacagggaaaccggatggcttgaaccatcaagttactcaggggaagaagagtcacactcagagtcaacagactggtccaacgacactgacttctgacaggtttcttgaCAGTTGTTTCATATGAAGAGATTGTTAAGTCACGATAATC‑AATAGGAGAAATCAATATGATCGTTTCTGACATCGAAGCTAACGCCCTCTTAGAGAGCGTCACTAAGTTCCACTGCGGGGTTATCTACGACTACTCCACCGCTGAGTACGTAAGCTACCGTCCGAGTGACTTCGGTGCGTATCTGGATg                                                                                                                                                    <  1:440707‑M2/189‑1 (MQ=255)`  
                                                                                                                                                           `acaacaaggaaaccggatggcttgaaccatcaagttactcaggggaagaagagtcacactcagagtcaacagactggtccaacgacactgacttctgacaggattcttgaCAGTTGTTTCATATGAAGAGATTGTTAAGTCACGATAATC‑AATAGGAGAAATCAATATGATCTTTTCGGACATCGAAGCTAACGCACTCTTCGAGAGCGTCACTAAGTTCCACTGCGGGGTTATCTACGACTACTCCACCGCAGAGTACGTAAGCTACCGTCCGAGTAACTTCGTTGCGTCTATGGATgcg                                                                                                                                                  >  1:65170‑M2/111‑301 (MQ=255)`  
                                                                                                                                                                 `aggaaaccggatggcttgaaccatcaagttactcaggggaagaagagtcacactcagagtcaacagactggtccaacgacactgacttctgacaggattcttgaCAGTTGTTTCATATGAAGAGATTGTTAAGTCACGATAATC‑AATAGGAGAAATCAATATGATCGTTTCTGACATCGAAGCTAACGCCCTCTTAGAGAGCGTCACTAAGTTCCACTGCGAGGTTATCTACGACTACTCCACCGCTGAGTACGTAAGCTACCGTCCGAGTGACTTCGGTGCGTATCTGGATGCGCTGGaa                                                                                                                                            >  1:407281‑M2/105‑301 (MQ=255)`  
                                                                                                                                                                      `gtgtataagagacagccatcaagttactcaggggaagaagagtcacactcagagtcaacagactggtccaacgacactgacttctgacaggattcttgaCAGTTGTTTCATATGAAGAGATTGTTAAGTCACGATAATC‑AATAGGAGAAATCAATATGATCGTTTCTGACATCGAAGCTAACGCCCTCTTAGAGAGCGTCACTAAGTTCCACTGCGGGGTTATCTACGACTACTCCACCACTGAGTACGTAAGCTACCGTCCGAGTGACTTCGGTGCGTATCTGGATGCGCTGGAAGCcgt                                                                                                                                       <  1:469032‑M2/202‑2 (MQ=255)`  
                                                                                                                                                                                  `agagacagaagttactcaggggaagaagagtcacactcagagtcaacagactggtccaacgacactgacttctgacaggattcttgaCAGTTGTGTCATATGAAGATATTGTTAAGTCACGATAATC‑AATAGGAGAAATCAATATGTTCGTTTCTGACATCGAAGCTAACGCCCTCTTAGAGAGCGTCACTAAGTTCCACTGCGGGGTTATCTACGACTACTCCACCGCTGAGTACGTAAGCTACCGTCCGCGTGACTTCGGTGCGTATCTGGATGCGCTGGAAGCCGAGGTTGCACGAgg                                                                                                                           <  1:145171‑M2/214‑1 (MQ=255)`  
                                                                                                                                                                                          `aagttactcaggggaagaagagtcacactcagagtcaacagactggtccaacgacactgacttctgacaggattcttgaCAGTTGTTTCATATGAAGAGATTGTTAAGTCACGATAATC‑AATAGGAGAAATCAATATGATCGTTTCTGACATCGAAGCTAACGCCCTCTTAGAGAGCGTCACTAAGTTCCACTGCGGGGTTATCTACGACTACTCCACCGCTGAGTACGTAAGCTACCGTCCGAGTGACTTCGGTGCGTATCTGGATGCGCTGGAAGCCGAGGTTGCACTAGGCGGTcttt                                                                                                                   >  1:342766‑M2/80‑300 (MQ=255)`  
                                                                                                                                                                                             `ttactcaggggaagaagagtcacactaagagtcaacagactggtccaacgacactgacttctgacaggattcttgaCAGTTGTTTCATATGAAGAGATTGTTAAGTCACGATAATC‑AATAGGAGAAATCAATATGATCGTTTCTGACATCGAAGCTAACGCCCTCTTAGAGAGCGTCACTAAGTTCCACTGCGGGGTTATCTACGACTACTCCACCGCTGAGTACGTAAGCTACCGTCCGAGTGACTTCGGTGCGTATCTGGATGCGCTGGAAGCCGAGGTTGCACGAGGCGGTCTTattt                                                                                                                >  1:153912‑M2/77‑300 (MQ=255)`  
                                                                                                                                                                                             `gagacagggggaagaagagtcacactcagaggcaacagactggtccaacgacactgacttctgacaggattcttgaCAGTTGTTTCATATGAAGAGATTGTTAAGTCACGATAATC‑AATAGGAGAAATCAATATGATCGTTTCAGACATCGAAGCTAACGCCCTCTTAGAGAGCGTCACTAAGTTCCACTGCGGGGTTATCTACGACTACTCCACCGCTGAGTACGTAAGCTACCGTCCGAGTGACTTCGGTGCGTATCTGGATGCGCTGGAAGCCGAGGTTGCACGAGGCGGTCTTATtg                                                                                                                <  1:217995‑M2/225‑1 (MQ=255)`  
                                                                                                                                                                                             `ctcggagatgtgtataagagacagctcagagtcaacagactggtccaacgacactgacttctgacaggattcttgaCAGTTGTTTCATATGAAGAGATTGTTAAGTCACGATAATC‑AATAGGAGAAATCAATATGATCGTTTCTGACATCGAAGCTAACGCCCTCTTAGAGAGCGTCACTAAGTTCCACTGCGGGGTTATCTACGACTACTCCACCGCTGAGTACGTAAGCTACCGTCCGAGTGACTTCGGTGCGTATCTGGATGCGCTTGAAGCCGAGGTTGCACGAGGCGGTCTTATtg                                                                                                                <  1:300844‑M2/225‑1 (MQ=255)`  
                                                                                                                                                                                               `actcaggggaagaagagtcacactcagagtcaacagactggtccaacgacactgacttctgacaggattcttgaCAGTTGTTTCATATGAAGAGATTGTTAAGTCACGATAATC‑AATAGGAGAAATCAATATGATCGTTTCTGACATCGAAGCTAACGCCCTCTTAGAGAGCGTCACTAAGTTCCACTGCGGGGTTATCTACGACTACTCCACCGCTGAGTACGTAAGCTACCGTCCGAGTGACTTCGGTGCGTATCTGGATGCGCTGGAAGCCGAGGTTGCACGAGGCGTGCTTattgct                                                                                                              >  1:48138‑M2/75‑299 (MQ=255)`  
                                                                                                                                                                                               `actcaggggaagaagagtcacactcagagtcaacagactggtccaacgacactgacttctgacaggattcttgaCAGTTGTTTCATATGAAGAGATTGTTAAGTCACGATAATC‑AATAGGAGAAATCAATATGATCGTTTCTGACATCGAAGCTAACGCCCTCTTAGAGAGCGTCACTAAGTTCCACTGCGGGGTTATCTACGACTACTCCACCGCTGAGTACGTAAGCTACCGTCCGAGTGACTTCGGTGCGTATCTGGATGCGCTGGAAGCCGAGGTTGCACGAGGCGGTCTTATtgtg                                                                                                              <  1:266997‑M2/227‑1 (MQ=255)`  
                                                                                                                                                                                                          `agtgtataaagagacaggtcaacagactggtccaacgacactgacttctgacaggattcttgaCAGTTGTTTCAGATGAAGAGATTGTTAAGTCACGATAATC‑AATAGGAGAAATCAATATGATCGTTTCTGACATCGAAGCTAACGCCCTCTTAGAGAGCGTCACTAAGTTCCACTGCGGGGTTATCTACGACTACTCCACCGCTGAGTACGTAAGCTACCGTCCGAGTGACTTCGGTGCGTATCTGGATGCGCTGGAAGCCGAGGTTGCACGAGGCGGTCTTATTGTGTTCCACAACgg                                                                                                   <  1:452095‑M2/238‑1 (MQ=255)`  
                                                                                                                                                                                                               `gtcacactcagagtcaacagactggtccaacgacactgacttctgacaggattcttgaCAGTTGTTTCATATGAAGAGATTGTTAAGTCACGATAATC‑AATAGGAGAAATCAATATGATCGTTTCTGACATCGAAGCTAACGCCCTCTTAGAGAGCGTCACTAAGTTCCACTGCGGGGTTATCTACGACTACTCCACCGCTGAGGACGTAAGCAACCGTCCGAGTGACTTCGGTGCGTATCTGGATGCTCTGGAAGCCGAGGTTGCACGAGGCGGTCTTATTGTGTTCCACAACGGTcaca                                                                                              >  1:285635‑M2/59‑301 (MQ=255)`  
                                                                                                                                                                                                               `ggggtataagagacagacagactggtccaacgacactgacttatgacaggattcttgaCAGTTGTTTCATATGAAGAGATTGTTAAGTCACGATAATC‑AATAGGAGAAATCAATATGATCGTTTCTGACATCGAAGCTAACGCCCTCTTAGAGAGCGTCACTAAGTTCCACTGCGGGGTTATCTACGACTACTCCACCGCTGAGTACGTAAGCTACCGTCCGAGTGACTTCGGTGCGTATCTGGATGCGCTGGAAGCCGAGGTTGCACGAGGCGGTCTTATTGTGTTCCACAACGGTcaca                                                                                              <  1:108876‑M2/243‑1 (MQ=255)`  
                                                                                                                                                                                                               `aCTCCACGCGGTGCAATCGTTGCCGATAAGACCAACATGATTCACACAGGTTTCTTGACAGTTGTTTCATATGAAGAGATTGTTAAGTCACGATAATC‑AATAGGAGAAATCAATATGATCGTTTCTGACATCGAAGCTAACGCCCTCTTAGAGAGCGTCACTAAGTTCCACTGCGGGGTTATCTACGACTACTCCACCGCTGAGTACGTAAGCTACCGTCCGAGTGACTTCGGTGCGTATCTGGATGCGCTGGAAGCCGAGGTTGCACGAGGCGGTCTTATTGTGTTCCACAACGGTcaca                                                                                              >  1:100278/1‑301 (MQ=255)`  
                                                                                                                                                                                                                 `cacactcagagtcaacagactggtccaacgaagctgacttctgacaggaatattgaCAGTTGATTCATATGAAGAAATTGTTAAGTTACGATAAAC‑AATAGTAGAAATCAATATGATCGTTTCTAACATTGAAGCTAACGCCCTCTTAGAGAGCGTCACTAAGTTCCACTGCGGGGTTATCTACGACTACTCCACCGCTGAGTACGTAAGCTACCGTCCTAGTCACTTCGGTGCGTATCTGGATGCGCTGGAAGCAGAGGTTGCACTAGGAGGTCTTATTGTGTTCCACAACGGTCACAAg                                                                                            <  1:509660‑M2/245‑1 (MQ=255)`  
                                                                                                                                                                                                                     `ctcagagtcaacagactggtccaacgacactgacttctgacaggattcttgaCAGTTGTTCCATATGAAGAGATTGTTAAGTCACGATAATC‑AATAGGAGAAATCAATATGATCGTTTCTGACATCGAAGCTAACGCCCTCTTAGAGAGCGTCACTAAGTTCCACTGCAGGGTTATCTACGACTACTCCACCGCTGAGTACGTAAGCTACCGTCCGAGTGACTTTGGTGCGTATCTGGATGCGCTGGAAGCCGAGGTTGCACGAGGCGGTCTTATTGTGTTCCACAACGGTCACAAGTATg                                                                                        >  1:269939‑M2/53‑301 (MQ=255)`  
                                                                                                                                                                                                                        `agattcaccagactggtccaacgacactgacttctgacaggatgcttgaCAGTTGTTTCATATGAAAAGATTGTTAAGTCACGATCATC‑AATAGGAGAAATCAATATGATCGTGTCTGACATCGAAGCTAACGCCCTCTTAGAGAGCGTCACTAAGTTCCACTTCGGGGTTATCTACGACTACTCCACCGTTGATTACGTAAGCTACCGTCCGAGTGACTTCGGTGCGTATCTGGATGCGCTGTAAGCCGAGGTTGCACGAGGCGGTCTTATTGTGTTCCACAACGGTCACAAGTATGACg                                                                                     <  1:48577‑M2/252‑1 (MQ=255)`  
                                                                                                                                                                                                                         `gagtcaacagactggtccaacgacactgacttctgacaggattcttgaCAGTTGTTTCATATGAAGAGATTGTTAAGTCACGATAATC‑AATAGGAGAAATCAATATGATCGTTTCTGACATCGAAGCTAACGCCCTCTTAGAGAGCGTCACTAAGTTCCACTGCGGGGTTATCTACGACTACTCCACCGCTGAGTATGTAAGCTACCGTCCGAGTGACTTCGGTGCGTATCTGGATGCGCTGGAAGCCGAGGTTGCACGAGGCGGTCTTATTGTGTTCCACAACGGTCACAAGTATGACGt                                                                                    <  1:29525‑M2/253‑1 (MQ=255)`  
                                                                                                                                                                                                                          `agtcaacagactggtccaacgacacggacttctgacaggattcttgaCAGTTGTTTCATATGAAGGGATTGTTAAGTCACGATAATC‑AATAGGAGAAATCAATATGATCGTTTCTGACATCGAAGCTAACGCACTCTTAGAGAGCGTCACTAAGTTCCACTGCGGGGTTATCTACGACTACTCCACCGATGAGTACGTAAGCTACCGTCCGAGTGACTTCGGTGCGTATCTGGATGCGCTGGAAGCCGAGGTTGCACGAGGCGGTCTTATTGTGTTCCACAACGGTCACAAGTATGACGtt                                                                                   <  1:117492‑M2/254‑1 (MQ=255)`  
                                                                                                                                                                                                                                 `agacttgtccaacgacaatgacttctgacaggattcttgaCAGTTGTTTCATATGAAGAGATTGTTAAGTCACGATAATC‑AATAGGAGAAATCAATATGATCGTTTCTGACATCGAAGCTAACGCCCTCTTAGAGAGCGTCACTAAGTTCCACTGCGGGGTTATCTACGACTACTCCACCGCTGAGTACGTAAGCTACCGTCCGAGTGACTTCGGTGCGTATCTGGATGCGCTGGAAGCCGAGGTTGCACGAGGCGGTCTTATTGTGTTCCACAACGGTCACAAGTATGACGTTACTGCAt                                                                            <  1:504430‑M2/261‑1 (MQ=255)`  
                                                                                                                                                                                                                                 `aaacaggtccaacgacactgacttctgacaggattcttgaCAGTTGTTTCATATGAAGAGATTGTTAAGTCACGATAATC‑AATAGGAGAAATCAATATGATCGTTTCTGACATCGAAGCTAACGCCCTCTTAGAGAGCGTCACTACGTTCCACTGCGGGGTTATCTACGACTACTCCACCGCTGAGTACGTAAGCTACCGTCAGAGTGACTTCGGTGCGTATCTGGCTGCGCTGGAAGCCGAGGTTGCACGAGGCGGTCTTATTGTGTTCCACAACGGTCACAAGTATGACGTTCCTGCAt                                                                            <  1:77448‑M2/261‑1 (MQ=255)`  
                                                                                                                                                                                                                                     `gtgtccaacgacactgacttctgacaggattcttgaCAGTTGTTTCATATGAAGAGATTGTTAAGTCACGATAATC‑AATAGGAGAAATCAATATGATCGTTTCTGACATCGAAGCTAACGCCCTCTTAGAGAGCGTCACTAAGTTCCACTGCGGGGTTATCTACGACTACTCCACCGCTGAGTACGTAAGCTACCGTCCGAGTGACTTCGGTGCGTATCTGGATGCGCTGGAAGCCGAGGTTGCACGAGGCGGTCTTATTGTGTTCCACAACGGTCACAAGTATGACGTTCCTGCATTGAc                                                                        >  1:364883‑M2/37‑301 (MQ=255)`  
                                                                                                                                                                                                                                      `ggtccaacgacactgacgtctgacaggattcttgaCAGGTGTTTCATATGAAGAGATTGTTAAGTCACGATAATC‑AATATGAGAAATCAATATGATCGTTTCTGACATCGAAGCTAACGCCCTCTTAGAGAGCGTCACTAAGTTCCACTGCGGGGTTATCTACGACTACTCCACCGCTGAGTACGTAAGCTACCGTCCGAGTGACTTCGGTGCGTATCTGGATGCGCTGGAAGCCGAGGTTGCACGAGGCGGTCTTATTGTGTTCCACAACGGTCACAAGTATGACGTTCCTGCATTGAcc                                                                       <  1:140497‑M2/266‑1 (MQ=255)`  
                                                                                                                                                                                                                                                  `aaTGATTCACACAGGTTTCTTGACAGTTGTTTCATATGAAGAGATTGTTAAGTCACGATAATC‑AATAGGAGAAATCAATATGATCGTTTCTGACATCGAAGCTAACGCCCTCTTAGAGAGCGTCACTAAGTTCCACTGCGGGGTTATCTACGACTACTCCACCGCTGAGTACGTAAGCTACCGTCCGAGTGACTTCGGTGCGTATCTGGATGCGCTGGAAGCCGAGGTTGCACGAGGCGGTCTTATTGTGTTCCACAACGGTCACAAGTATGACGTTCCTGCATTGACCAAACTGGCAAAg                                                           <  1:198103/300‑1 (MQ=255)`  
                                                                                                                                                                                                                                                   `gtccaacgacactgacttctgaCAGTTGTTTCATATGAAGAGATTGTTAAGTCACGATAATC‑AATAGGAGAAATCAATATTATCGTTTCTGACATCGAAGCTAACGCCCTCTTAGAGAGCGTCACTAAGTTCCACTGCGGGGTTATCTACGACTACTCCACCGCTGAGTACGTAAGCTACCGTCCGAGTGACTTCGGTGCGTATCTGGATGCGCTGGAAGCCGAGGTTGCACGAGGCGGTCTTATTGTGTTCCACAACGGTCACAAGTATGACGTTCCTGCATTGACCAAACTGGCAAAGt                                                          >  1:91382‑M2/23‑301 (MQ=255)`  
                                                                                                                                                                                                                                                     `gATTCACACATGTTTCTTGACAGTTGTTTCATATGAAGAGATTGTTAAGTCACGATAATC‑AATAGGAGAAATCAATATGATCGTTTCTGACATCGAAGCTAACGCCCTCTTAGAGAGCGTCACTAAGTTCCACTGCGGGGTTATCTACGACTACTCCACCGCTGAGTACGTAAGCTACCGTCCGAGTGACTTCGGTGCGTATCTTGATGCGCTGGAATCCGAGGTTGCACGAGGCGGTCTTATTGTGTTCCACAACGGTCACAAGTATGACGTTCCTGCATTGACCAAACTGGCAAAGtgg                                                        >  1:411949/1‑299 (MQ=255)`  
                                                                                                                                                                                                                                                      `cttctgacaggattcttgaCAGTTGTTTCATATGAAGAGATTGTTAAGTCACGATAATC‑AATAGGAGAAATCAATATGATCGTTTCTGACATCGAAGCTAACGCCCTCTTAGAGAGCGTCACTAAGTTCCACTGCGGGGTTATCTACGACTACTCCACCGCTGAGTACGTAAGCTACCGTCCGAGTGACTTCGTTGCGTATCTGGATGCGCTGGAAGCCGAGGTTGCACGAGGCGGTCTTATTGTGTTCCACAACGGTCACAAGTATGACGTTCCTGCATTGACCAAACTGGCAAAGTTGc                                                       >  1:358686‑M2/20‑301 (MQ=255)`  
                                                                                                                                                                                                                                                               `ggattcttgaCAGTTGTTTCATATGAAGAGATTGTTAAGTCACGATAATC‑AATAGGAGAAATCAATATGATCGTTTCTGACATCGAAGCTAACGCCCTCTTAGAGAGCGTCACTAAGTTCCACTGCGGGGTTATCTACGACTACTCCACCGCTGCGTACGTACGCTACCGTCCGAGTGACTTCGGTGCGTATCTGGATGCGCTGGAAGCCGAGGTTGCACGAGGCGGTCTTATTGTGTTCCACAACGGTCACAAGTATGACGATCCTGCATTGACCACACTGGCAAAGTTGCAATTGAact                                              >  1:132162‑M2/11‑300 (MQ=255)`  
                                                                                                                                                                                                                                                                `gattcttgaCAGTTGTTTCATATGAAGAGATTGTTAAGTCACGATAATC‑AATAGGAGAAATCAATATGATCGTTTCTGACATCGAAGCTAACGCCCTCTTAGAGAGCGTCACTAAGTTCCACTGCGGGGTTATCTACGACTACTCCACCGCTGCGTACGTAAGCTACCGTCCGAGTGACTTCGGTGCGTATCTGGATGCGCTGGAAGCCGAGGTTGCACGAGGCGGTCTTATTGTGTTCCACAACGGTCACAAGTATGACGTTCCTGCATTGACCTAACTGGCAAAGTTGCAATTGAACCg                                             >  1:428958‑M2/10‑301 (MQ=255)`  
                                                                                                                                                                                                                                                                    `cttgaCAGTTGTTTCATATGAAGAGATTGTTAAGTCACGATAATC‑AATAGGAGAAATCAATATGATCGTTTCTGACATCGAAGCTAACGCCCTCTTAGAGAGCGTCACTAAGTTCCACTGCGGGGTTATCTACGACTACTCCACCGCTGAGTACGTAAGCTAACGTCCGAGTGACTTCGGTGCGTATCTGGATGCGCTGGAAGCCGAGGTTGCACGAGGCGGTCTTATTGTGTTCCACAACGGTCACAAGTTTGACGTTCCTGCATTGACCAAACTGGCACAGTTGCAATTGAACCGagag                                         >  1:372343‑M2/6‑301 (MQ=255)`  
                                                                                                                                                                                                                                                                           `gttgttTCATATGAAGAGATTGTTAAGTCACGATAATC‑AATAGGAGAAATCAATATGATCGTTTCTGACATCGAAGCTAACGCCCTCTTAGAGAGCGTCACTAAGTTCCACTGCGGGGTTATCTACGACTACTCCACCGCTGAGTACGTAAGCTACCGTCCGAGTGACTTCGGTGCGTATCTGGATGCGCTGGAAGCCGAGGTTGCACGAGGCGGTCTTATTGTGTTCCACAACGGTCACAAGTATGACGTTCCTGCATTGACCAAACTGGCAAAGTTGCAATTGAACCGAGAGTTCCAcc                                  <  1:235924/301‑1 (MQ=255)`  
                                                                                                                                                                                                                                                                               `tttCATATGAAGAGATTGTTAAGTCACGATAATC‑AATAGGAGAAATCAATATGATCGTTTCTGACATCGAAGCTAACGCCCTCTTAGAGAGCGTCACTAAGTTCCACTGCGGGGTTATCTACGACTACTCCACCGCTGAGTACGTAAGCTACCGTCCGAGTGACTTCGGTGCGTATCTGGATGCGCTGGAAGCCGAGGTTGCACGAGGCGGTCTTATTGTGTTCCACAACGGTCACAAGTATGACGTTCCTGCATTGACCAAACTGGCAAAGTTGCAATTGAACCGAGAGTTCCACCTTcc                              <  1:367328/301‑1 (MQ=255)`  
                                                                                                                                                                                                                                                                                  `cATATGAAGAGATTGTTAAGTCACGATAATC‑AATAGGAGAAATCAATATGATCGTTTCTGACATCGAAGCTAACGCCCTCTTAGAGAGCGTCACTAAGTTCCACTGCGGGGTTATCTACGACTACTCCACCGCTGAGTACGTAAGCTACCGTCCGAGTGACTTCGGTGCGTATCTGGATGCGCTGGAAGCCGAGGTTGCACGAGGCGGTCTTATTGTGTTCCACAACGGTCACAAGTATGACGTTCCTGCATTGACCAAACTGGCAAAGTTGCAATTGAACCGAGAGTTCCACCTTCCTCg                           <  1:460191/301‑1 (MQ=255)`  
                                                                                                                                                                                                                                                                                  `ataagagacagaTTGTTAAGTCACGATAATC‑AATAGGAGAAATCAATATGATCGTTTCTGACATCGAAGCTAACGCCCTCTTAGAGAGCGTCACTAAGTTCCACTGCGGGGTTATCTACGACTACTCCACCGCTGAGTACGTAAGCTACCTTCCGAGTGACTTCGGTGCGTATCTGGATGCGCTGGAAGCCGAGGTTGCACGATGCGGTCTTATTGTGTTCCACAACGGTCACAAGTATGACGTTCCTGCATTGACCAAACTGGCAAAGTTGCAATTGAACCGAGAGTTCCACCTTCCTCg                           <  1:149195/292‑1 (MQ=255)`  
                                                                                                                                                                                                                                                                                    `tatGAAGAGATTGTTAAGTCACGATAATCAAACAGGAGAAACCATTATGATCGTTTCTGACATCGAAGCTAACGCCCTCTTAGAGAGCGTCACTAAGTTCCACTGCGGGGTTATCTACGACTACTCCACCGATGAGTACGTAAGCTACCGTCCGAGTGACTTCGGTGCGTATCTGGATGCGCTGGAAGCCGAGGTTGCACGAGGCGGTCTTATTGTGTTCCACAACGGTCACAAGTATGACGTTCCTGCATTGACCAAACTGGCAAAGTTGCAATTGAACCGAGAGTTCCACCTTCCTCGt                          >  1:45559/1‑301 (MQ=255)`  
                                                                                                                                                                                                                                                                                    `tatGAAGAGATTGTTAAGTAACGATAATC‑AATAGGAGAAATCAATATGATCGTTTCTGACATCGAAGCTAAAGCCCTCTTAGAGAGCGTCACTAAGTTCCACTGCGGGGTTATCCATGACTACTCCACCGCGGAGTACGTAAGCTACCGTCCGAGTGACTTCGGTGCGTATCTGGATGCGATGGCAGCCGAGGTTGCACGAGGCGGTCTTATTTTGTTCCACAACGGTAACAAGCATGCAGTTCCTGCATTGCCCCAACTGGCAAAGTTGCAATTGAACCGAGAGTTCCACCTTCATAGTg                         >  1:315899/1‑301 (MQ=255)`  
                                                                                                                                                                                                                                                                                    `cgtaaagaggccacacaGTCACGATAATC‑AATAGGAGAAATCAATATGATCGTTTCTGACATCGAAGCTAACGCCCTCTTAGAGAGCGTCACTAAGTTCCACTGCGGGGTTATCTACGACTACTCCACCGCTGAGTACGTAAGCTACCGTCCGAGTGACTTCGGTGCGTATCTGGATGCGCTGGAAGCCGAGGTTGCACGAGGCGGTCTTATTGTGTTCCACAACGGTCACAAGTATGACGTTCCTGCATTGACCAAACTGGCAAAGTGGCAATTGAACCGAGAGTTCCACCtgtctctta                         >  1:68848/17‑293 (MQ=255)`  
                                                                                                                                                                                                                                                                                      `tGAAGAGATTGTTAAGTCACGATAATC‑AATAGGAGAAACCATTATGATCGTTTCTGACATCGAAGCTAACGCCCTCTTAGAGAGCGTCACTAAGTTCCACTGCGGGGTTATCTACGACTACTCCACCGCTGAGTACGTAAGCTACCGTCCGAGTGACTTCGGTGCGTATCTGGATGCGCTGGAAGCCGAGGTTGCACGAGGCGGTCTTATTGTGTTCCACAACGGTCACAAGTATGACGTTCCTGCATTGACCAAACTGGCAAAGTTGCAATTGAACCGAGAGTTCCACCTTCCTCGTgag                       >  1:276378/1‑301 (MQ=255)`  
                                                                                                                                                                                                                                                                                         `agagATTGTTAAGTCACGATAATC‑AATAGGAGAAATCAATATGATCGTTTCTGACATCGAAGCTAACGCCCTCTTAGAGAGCGTCACTAAGTTCCACTGCGGGGTTATCTACGACTACTCCACCGCTGAGTACGTAAGCTACCGTCCGAGTGACTTCGGTGCGTATCTGGATGCGCTGGAAGCCGAGGTTGCACGAGGCGGTCTTATTGTGTTCCACAACGGTCACAAGTATGACGTTCCTGCATTGACCAAACTGGCAAAGTTGCAATTGAACCGAGAGTTCCACCTTCCTCGTGAGAAc                    <  1:309973/301‑1 (MQ=255)`  
                                                                                                                                                                                                                                                                                         `agagATTGTTAAGTCACGATAATC‑AATAGGAGAAATCAATATGATCGTTTCTGACATCGAAGCTAACGCCCTCTTAGAGAGCGTCACTAAGTTCCACTGCGGGGTTATCTACGACTACTCCACCGCTGAGTACGTAAGCTACCGTCCGAGTGACTTCGGTGCGTATCTGGATGCGCTGGAAGCCGAGGTTGCACGAGGCGGTCTTATTGTGTTCCACAACGGTCACAAGTATGACGTTCCTGCATTGACCAAACTGGCAAAGTTGCAATTGAACCGAGAGTTCCACCTTCCTCGTGAGAAc                    <  1:73603/301‑1 (MQ=255)`  
                                                                                                                                                                                                                                                                                         `acggaTTCTGAAGTACCGATAAGT‑AGTAGGAGAAATCAATATGCTCGTTTCTGACATTGAATGTAAGGGACTCTTCGAGAGCGTCACTAAGTTCTACTGCTGGGTTATCTACGACTACTCCACCGCTGAGTACGTAAGCTACCGGCCGAGTGACTTCGGTGCGTATCTGGATGCGCTGGAAGACGAGGTTGCACGAGGCGGTCTTATTGTGTTCCACAACGGTCACAAGTATGACGTTGCTGCATTGACCAAACTTGCAAAGTTGCAATTGAACCGAGAGTTCCACCTTCCTCGTGAGAAc                    <  1:497626/298‑1 (MQ=255)`  
                                                                                                                                                                                                                                                                                            `gaTTGTTAAGTCACGATAATC‑AATAGGAGAAATCAATATGATCGTTTCTGACATCGAAGCTAACGCCCTCTTAGAGAGCGTCACTAAGTTCCACTGCGGGGTTATCTACGACTACTCCACCGCTGAGTACGTAAGCTCCCGTCCGAGTGACTTCGGTGCGTATCTGGATGCGCTGGAAGCCGAGGTTGCACGAGGCGGTCTTATTGTGTTCCACAACGGTCACAAGTATGACGTTCCTGCATTGACCAAACTGGCAAAGTTGCAATTGAACAGAGAGTTCCACCTTCCTAGTGAGAACTGt                 >  1:232005/1‑301 (MQ=255)`  
                                                                                                                                                                                                                                                                                                `gTTAAGTCGCGATAATC‑AATAGGAGAAATCAATATGATCGTTTCTGACATCGAAGCTAACGCCCTCTTAGAGAGCGTCACTAAGTTCCACTGCGGGGTTATCTACGACTACTCCACCGCTGAGTACGTAAGCTACCGTCCGAGTGACTTCGCTGCGTATCTGGATGCGCTGGAAGCCGAGGTTGCACGAGGCGGTCTTATTGTGTTCCACAACGGCACCAAGAATGACGTTCCTCCATTGACCAAACTGGCACAGTTGCAATCTAACCGAGACTTTCCCGTTCCTCGTGTGAACTGTAttt             >  1:462237/1‑300 (MQ=255)`  
                                                                                                                                                                                                                                                                                                  `tAAGTCACGATAATC‑AATAGGAGAAATCAATATGATCGTTTCTGACATCGAAGCTAACGCCCTCTTAGAGAGCGTCACTAAGTTCCACTGCGGGGTTATCTACGACTACTCCACCGCTGAGTACGTAAGCTACCGTCCGAGTGACTTCGGTGCGTATCTGGATGCGCTGGAAGCCGAGGTTGCACGAGGCGGTCTTATTGTGTTCCACAACGGTCACAAGTATGACGTTCCTGCATTGACCAAACTGGCAAAGTTGCAATTGAACCGAGAGTTCCACCTTCCTCGTGAGAACTGTATTGac           <  1:18479/301‑1 (MQ=255)`  
                                                                                                                                                                                                                                                                                                   `aaGTCACGATAATC‑AATAGGAGAAATCAATATGATCGTTTCTGACATCGAAGCTAACGCCCTCTTAGAGAGCGTCACTAAGTTCCACTGCGGGGTTATCTACGACTACTCCACCGATGAGTACGTAAGCTACCGTCCGAGTGACTTCGGTGCGTATCTGGATGCGCTGGAAGCCGAGGTTGCACGAGGCGGTCTTATTGTGTTCCACAACGGTCACAAGTATGACGTTCCTGCATTGACCAAACTGGCAAAGTTGCAATTGAACCGAGAGTTCCACCTTCCTCGTGAGAACTGTATTGaca          <  1:348625/301‑1 (MQ=255)`  
                                                                                                                                                                                                                                                                                                    `aGTCACGATAATC‑AATAGGAGAAATCAATATGATCGTTTCTGAAATCGAAGCTAACGCCCTCTTAGAGAGCGTCACTAAGTTCCACTGCGGGGTTATCTACGACTACTCCACCGCTGAGTACGTAAGCTACCGTCCGAGTGACTTCGGTGCGTATCTGGATGCGCTGGAAGCCGAGGTTGCACGAGGCGGTCTTATTGTGTTCCACAACGGTCACAAGTATGACGTTCCTGCATTGACCAAACTGGCAAAGTTGCAATTGAACCGAGAGTTCCACCTTCCTCGTGAGAACTGTATTGacac         <  1:179954/301‑1 (MQ=255)`  
                                                                                                                                                                                                                                                                                                     `gTCACGATAATC‑AATAGGAGAAATCAATATGATCGTTTCTGACATCGAAGCTAACGCCCTCTTAGAGAGCGTCACTAAGTTCCACTGCGGGGTTATCTACGACTACTCCACCGCTGAGTACGTAAGCTACCGTCCGAGTGACTTCGGTGCGTATCTGGATGCGCTGGAAGCCGAGGTTGCACGAGGCGGTCTTATTGTGTTCCACAACGGTCACAAGTATGACGTTCCTGCATTGACCAAACTGGCAAAGTTGCAATTGAACCGATAGTTCCACCTTCCTCGTGAGAACTGTATTGACAcc        >  1:29036/1‑301 (MQ=255)`  
                                                                                                                                                                                                                                                                                                     `gTCACGATAATC‑AATAGGAGAAATCAATATGATCGTTTCTGACATCGAAGCTAACGCCCTCTTAGAGAGCGTCACTAAGTTCCACTGCGGGGTTATCTACGACTACTCCACCGCTGAGTACGTAAGCTACCGTCCGAGTGACTTCGGTGCGTATCTGGATGCGCTGGAAGCCGAGGTTGCACGAGGCGGTCTTATTGTGTTCCACAACGGTCACAAGTATGACGTTCCTGCATTGACCAAACTGGCAAAGTTGCAATTGAACCGAGAGTTCCACCTTCCTCGTGAGAACTGTATTGACAcc        >  1:394568/1‑301 (MQ=255)`  
                                                                                                                                                                                                                                                                                                         `cGATAATC‑AATAGGAGAAATCAATATGATCGTTTCTGACATCGAAGCTAACGCCCTCTTAGAGAGCGTCACTAAGTTCCACTGCGGGGTTATCTACGACTACTCCACCGCTGAGTACGTGAGCTACCGTCCGAGTGACTTCGGTGCGTATCTGGATGCGCTGGAAGCCGAGGTTGCACGAGGCGGTCTTATTGTGTTCCACAACGGTAACAAGTATGACGTTCCTGCATTGACCAAACTGGCAAAGTTGCAATTAAACCGAGAGTTCCACCTTCCTCTTGAGAACTGTATTGACACCCttt    >  1:106909/1‑300 (MQ=255)`  
                                                                                                                                                                                                                                                                                                           `aTAATC‑AATAGGAGAAATCAATATGATCGTTTCTGACATCGAAGCTAACGCCCTCTTAGAGAGCGTCACTAAGTTCCACTGCGGGGTTATCTACGACTACTCCACCGCTGAGTACGTAAGCTACCGTCCGAGTGACTTCGGTGCGTATCTGGATGCGCTGGAAGCCGAGGTTGCACGAGGCGGTCTTATTGTGTTCCACAACGGTCACAAGTATGACGTTCCTGCATTGACCAAACTGGCAAAGTTGCAATTGAACCGAGAGTTCCACCTTCCTCGTGAGAACTGTATTGacctgtctctt  >  1:431970/1‑292 (MQ=255)`  

                                                                                                                                                                                                                                                                                                           `|`                                                                                                                                                                                                                                                                                                               
`AAGAACTTAGGCTGGACTTGGACAAGACAGACTGGCTGGAAGAAACCAGAAGTTACCAAGCTGAGTCATAAGGTGTTCGATAAGGACACTATGACCCACATCAAGGCTGGTGATTGGGTTAAGGTTGACATGGGAGTTGTTGGTGGATACGGCTACGTCCGCTCAGTTAGTGGCAAATATGCACAAGTGTCATACATCACAGGTGTTACTCCACGCGGTGCAATCGTTGCCGATAAGACCAACATGATTCACACAGGTTTCTTGACAGTTGTTTCATATGAAGAGATTGTTAAGTCACGATAATC‑AATAGGAGAAATCAATATGATCGTTTCTGACATCGAAGCTAACGCCCTCTTAGAGAGCGTCACTAAGTTCCACTGCGGGGTTATCTACGACTACTCCACCGCTGAGTACGTAAGCTACCGTCCGAGTGACTTCGGTGCGTATCTGGATGCGCTGGAAGCCGAGGTTGCACGAGGCGGTCTTATTGTGTTCCACAACGGTCACAAGTATGACGTTCCTGCATTGACCAAACTGGCAAAGTTGCAATTGAACCGAGAGTTCCACCTTCCTCGTGAGAACTGTATTGACACCCTTGTG  >  NC_001604.1/14032‑14631`

  

Alignment Legend

Aligned base mismatch/match (shaded by quality score): `ATCG/ATCG` `< 3 ≤` `ATCG/ATCG` `< 10 ≤` `ATCG/ATCG` `< 22 ≤` `ATCG/ATCG` `< 34 ≤` `ATCG/ATCG` `< 38 ≤` `ATCG/ATCG`

Unaligned base: `atcg`    Masked matching base: `atcg`    Alignment gap: `‑`    Deleted base: `‑`

Reads not counted as support for junction

read\_name Not counted due to insufficient overlap past the breakpoint.

read\_name Not counted due to not crossing MOB target site duplication.
